# Biologic Agents in Crohn's Patients Reduce CD4^+^ T Cells Activation and Are Inversely Related to Treg Cells

**DOI:** 10.1155/2022/1307159

**Published:** 2022-07-31

**Authors:** Eliane Aparecida Rosseto-Welter, Leticia D'argenio-Garcia, Filipa Blasco Tavares Pereira Lopes, Ana Eduarda Zulim Carvalho, Fernando Flaquer, Vanessa Severo-Lemos, Claudia Concer Viero Nora, Flavio Steinwurz, Lucas Pires Garcia Oliveria, Thiago Aloia, Luiz Vicente Rizzo, Cristóvão Luis Pitangueira Mangueira, Karina Inacio Carvalho

**Affiliations:** ^1^Hospital Israelita Albert Einstein, São Paulo, Brazil; ^2^Case Western Reserve University, Cleveland, Ohio, USA

## Abstract

Crohn's disease (CD) is a chronic inflammatory disease with a complex interface of broad factors. There are two main treatments for Chron's disease: biological therapy and nonbiological therapy. Biological agent therapy (e.g., anti-TNF) is the most frequently prescribed treatment; however, it is not universally accessible. In fact, in Brazil, many patients are only given the option of receiving nonbiological therapy. This approach prolongs the subsequent clinical relapse; however, this procedure could be implicated in the immune response and enhance disease severity. Our purpose was to assess the effects of different treatments on CD4^+^ T cells in a cohort of patients with Crohn's disease compared with healthy individuals. To examine the immune status in a Brazilian cohort, we analyzed CD4^+^ T cells, activation status, cytokine production, and Treg cells in blood of Crohn's patients. Patients that underwent biological therapy can recover the percentage of CD4^+^CD73^+^ T cells, decrease the CD4^+^ T cell activation/effector functions, and maintain the peripheral percentage of regulatory T cells. These results show that anti-TNF agents can improve CD4^+^ T cell subsets, thereby inducing Crohn's patients to relapse and remission rates.

## 1. Introduction

Crohn's disease (CD) is a chronic inflammatory disease of a complex interface with broad factors. CD is a dysfunction of environmental factors, genetic background, and microbiome dysregulation, resulting in an imbalance of the immune response. The first clinical manifestations in patients are weight loss, fatigue, chronic diarrhea, abdominal pain, and fever [1–4]. The incidence of CD is increasing worldwide, including in Brazil [5, 6]. Several types of treatment, biological and nonbiological (anti-inflammatory, antibiotics, corticoids, and glucocorticoids), have been shown to treat CD's symptoms [7]. In Brazil, patients commonly receive nonbiological treatments, whereas the most commonly used biological agent is anti-TNF alpha, but it is not widely accessible. Therefore, biological treatment is prescribed to patients with a higher disease risk to induce and maintain remission.

The immune response plays a pivotal role in the pathogenesis of Crohn's disease [8, 9]. Although, the patient's immune response has not been fully characterized. Many cells play different roles in the gut immune response. T cells are described as tissue-resident in the gut and can contribute to tissue damage or recovery during disease development. T regulatory (Treg) cells are a specialized subset of CD4^+^ T cells whose function depends on the transcription factor FOXP3 (forkhead box P3), essential for maintaining immune homeostasis [10]. Although, experimental models have shown that immune cells, such as Treg cells and CD8 T cells, can improve the immune response in colitis [11, 12]. Furthermore, maintenance of immunological tolerance in the intestinal mucosa is also achieved by Treg cells, suppressing immune activation [12]. In addition, the expression of CD39, an ectonucleotidase with coexpressing CD73, characterizes a specific subset of CD4^+^ Treg cells with augmented suppressor function [13].

Given the potential of anti-TNF agents to improve the remission of the majority of CD patients, we decided to investigate the impact of anti-TNF agents on the CD4^+^ T conventional, Treg, and suppressor Treg cells. Since all these subsets are important to maintaining homeostasis in the gut, we hypothesize that anti-TNF agents can recover these subsets, decrease inflammation, and improve the immune response of CD patients. Patients that underwent biological therapy can recover the percentage of CD4^+^CD73^+^ T cells, decrease CD4^+^ T cell activation/effector functions, and maintain the peripheral percentage of regulatory T cells. These results show that anti-TNF agents can improve CD4^+^ T cell subsets, thereby inducing Crohn's patients to relapse and remission rates.

## 2. Materials and Methods

### 2.1. Study Population

Our study cohort included three distinct groups: healthy, biological therapy, and nonbiological therapy. The healthy group is comprised of 28 individuals. None of these subjects had ever been diagnosed with any other chronic or autoimmune disease. We included 41 adult patients who had previously been diagnosed with CD according to the usual endoscopic, histological, and imaging criteria, and disease activity was measured using Crohn's disease activity index (CDAI). Of these, a total of 25 patients (median age of 36 years old) were treated with anti-TNF agents and 16 with nonbiological therapy (median age of 41 years old) (Table 1). All the subjects tested negative for HIV-1/2, HTLV-1/2, syphilis, HCV, and HBV. We also collected four tissue samples from patients who underwent surgery and a pathologist's report. The majority of the participants in the study were female. The Ethics Committee of the Hospital Israelita Albert Einstein approved this study. All samples were collected following informed written consent from all participants.

### 2.2. Flow Cytometry

Peripheral blood mononuclear cells (PBMCs) are isolated according to a well-established procedure and frozen in appropriate media until flow cytometry acquisition. Cells were stained for 30 min at room temperature followed by anti-CD3 (clone: SK3), anti-CD4 (clone: RPA-T4), anti-CD25 (clone: M-A251), anti-CD127 (clone: HLA-DR-M21), anti-CD39 (clone: TU66), anti-CD38 (clone: HB7), and anti-HLA-DR (clone: G46-6) from BD Biosciences; anti-CD73 (clone: AD2) from Biolegend; and amine aqua dye used for live-dead staining from ThermoFisher. After surface molecules were stained, cells were washed, fixed, and permeabilized (fixation–permeabilization buffer, eBioscience) for transcription factor anti-FOXP3 (clone: 259D/C7). For the analysis of cytokine production, intracellular PBMCs were incubated at 100 ng/ml alone or with phorbol 12-myristate 13-acetate (PMA) and 500 ng/mL of ionomycin. After 1 hour, monensin (5 mg/ml) and brefeldin A (5 mg/ml), both from BD Biosciences, were added to the culture. The cells were stained for the surface with anti-CD3 and anti-CD4 using amine aqua dye and incubated for 30 min at room temperature. Cells were washed, fixed, and permeabilized, following the manufacturer's instructions from Life Technologies. For intracellular cytokine production, we used anti-IL-17 (clone: BL28) from Biolegend and IFN-*γ* (clone: B27) and TNF-*α* (clone: Mab11) from BD Bioscience. Acquisition on a BD FACS Fortessa and analysis was done in FlowJo v10 (BD Bioscience) software.

### 2.3. Immunofluorescent Staining of Colon Section

Colon sections were cut 5 mm, and after heat-induced epitope retravel, cells were incubated overnight at 4°C with rabbit anti-human FoxP3 polyclonal antibody (Abcam). After washing, sections were stained with a secondary antibody anti-rabbit Alexa 568 (ThermoFisher) for two hours at room temperature. The sections were washed and incubated for two hours with the following antibodies: anti-human mouse CD4 (clone: RPA-T4), CD39 (clone: TU66), CD25 (clone: M-A251), and CD73 (clone: AD2) (BD Bioscience). Afterward, the sections were incubated with DAPI and mounted on slides using ProLong Gold antifade reagent (ThermoFisher). Images were acquired on the LSM 710 confocal microscope (Carl Zeiss) with a 20X objective. The colocalization was assessed using Zen 2012 SP2. The expression of CD4^+^CD25^+^FoxP3^+^CD39^+^CD73^+^ defined the Treg suppressor; for the Treg cells, the expression was used CD4^+^CD25^+^FoxP3^+^. At least ten different fields were counted for each sample.

### 2.4. Statistics

Statistical analysis was performed with GraphPad Prism version 7 (GraphPad Software, La Jolla, California, USA). The nonparametric Mann–Whitney test was used for all statistical comparisons and the Pearson correlation coefficient. All data were reported as medians with a 25–75% interquartile range (M; IQR); a *p* value of 0.05 or less was considered significant.

## 3. Results

### 3.1. Clinical Characteristics and Laboratory Features

Forty-one patients were treated with anti-TNF agents (21 with adalimumab and four with infliximab), and 16 patients treated with nonbiological therapy (Table 1) were analyzed. We also recruited 28 healthy subjects. These included the majority, around 70% of females and approximately 40 years old (Table 1).

### 3.2. Anti-TNF Therapy Increased CD4^+^CD73^+^ T Cells in the Periphery

**Figure 1:**
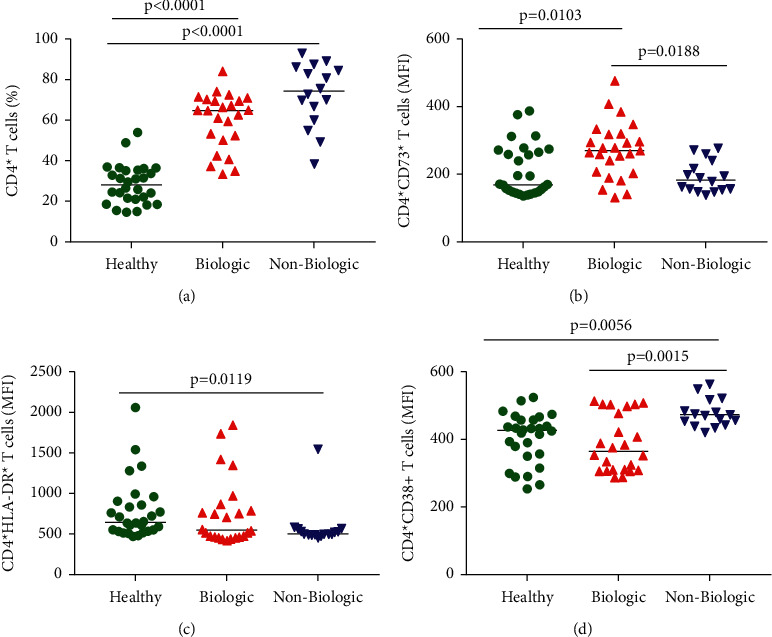
Phenotypic characterization of CD4^+^ T cells. Green plots healthy individuals, red triangles represent Crohn's patients undergoing biological therapy, and blue triangles represent Crohn's patients undergoing nonbiological therapy. (a) The subject's PBMC showing the percentage of total CD4^+^ T cells. (b)–(d) Median fluorescence intensity (MFI) of CD4^+^ T cells expressing CD73^+^, HLA-DR^+^, and CD38^+^, respectively. Each dot represents an individual, and bars indicate medians in the graphs. Statistical analysis was performed using the Kruskal–Wallis test.

In this cohort, the percentage of CD4^+^ T cells was elevated in both groups of patients (Figure 1(a)). T helper cells (CD4^+^ T cells) are essential to participate actively in the immune response and improve homeostasis in several diseases. Since we observe an increase of CD4^+^ T cells in the periphery, we ask if these cells will be related to activation markers. CD4^+^CD73^+^ T cells show an increase in the group of patients who use the biological therapy compared with the other groups (Figure 1(b)). The expression of CD73 alone by CD4^+^ T cells contributes to adenosine, which helps to activate the profile of CD4^+^CD73^+^ T cells. When we look at the fluorescence intensity for the CD4^+^HLA-DR^+^ marker, the biologic group had similar results to healthy subjects; however, the nonbiological group decreased the expression (Figure 1(c)). HLA-DR is important for T cell recognition and is used as a marker for T cell activation [14, 15]. On the other hand, traditional activation markers such as CD4^+^CD38^+^, which are used as a prognostic marker in several cases, show an increase in patients undergoing nonbiological treatment compared with the two other groups (Figure 1(d)).

### 3.3. Biologic Therapy Decreases CD4^+^ T Cells Phenotypic Characteristics of Memory and Th17

**Figure 2:**
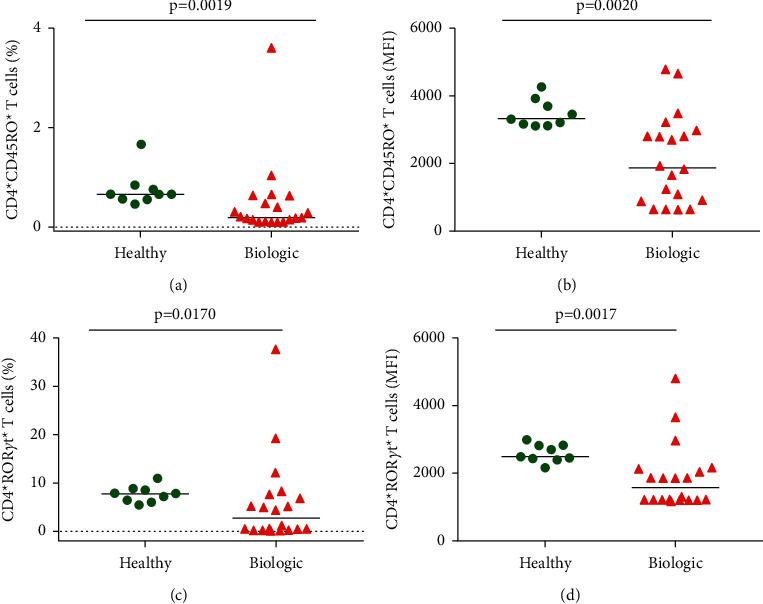
Characteristics of memory and effector markers on CD4^+^ T cells. Green plots healthy individuals and red triangles represent Crohn's patients undergoing biological therapy. (a) Percentage of CD4^+^ T cells expressing CD45RO^+^. (b) Median fluorescence intensity (MFI) of CD4^+^ T cells expressing CD45RO^+^. (c) Percentage of CD4^+^ T cells expressing ROR*γ*t^+^. (d) Median fluorescence intensity (MFI) of CD4^+^ T cells expressing RORgt+. Each dot represents an individual, and bars indicate medians in the graphs. Statistical analysis was performed using the Kruskal–Wallis test.

Since we observed a difference in activation markers expression, we investigated the CD45RO^+^ and the transcription factor ROR*γ*t^+^ expression on CD4^+^ T cells. Our results show that biologic therapy can decrease the expression of these markers (CD4^+^ CD45RO^+^ and CD4^+^ ROR*γ*t^+^) expression on CD4^+^ T cells, as shown in Figure 2. CD45RO is an important marker for the T cell memory profile, while ROR*γ*t denotes the T cell subpopulation related to inflammatory responses, such as the secretion of IL-17, IL-22, and others [16, 17].

### 3.4. Nonbiological Therapy Influences IL-17/Treg Cell Ratio

**Figure 3:**
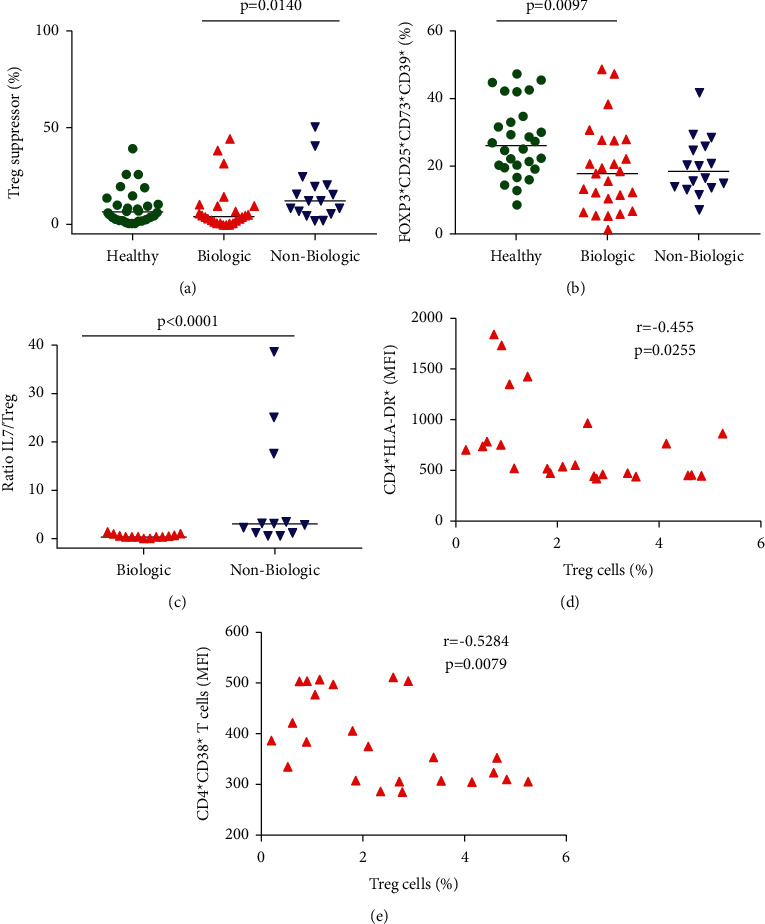
Biological therapy shows a lower percentage of CD4^+^ subsets of cells. Analysis was done previously on gating CD4^+^ T cells. (a) Frequencies of Treg suppressor cells on PBMCs. (b) Percentage of FOXP3^+^CD25^+^CD73^+^CD39^+^ in the groups. (c) The ratio of secretion of IL17 and expression of Treg in the groups of treated patients. Intracellular cytokine staining of PBMCs from a representative Crohn's patient after stimulation with PMA/ionomycin. (d)-(e) Correlation of percentage of natural Tregs and CD4^+^HLA-DR^+^ T cells and CD4^+^CD38^+^ T cells in Crohn's patients undergoing biological therapy, respectively. Each dot represents an individual, and bars indicate medians in the graphs. Statistical analysis was performed using the Kruskal–Wallis test and correlation Pearson test with alpha equal to 0.05.

To test biological therapy's ability to interfere in the Treg cells' expression in Crohn's disease patients, we investigated the natural and suppressor Treg. Unfortunately, we could not observe the difference in the natural Treg cells (CD3^+^CD4^+^CD25^high^CD127^−^FOXP3^+^) in all groups (data not shown). However, when we analyzed the characteristics of suppressor Treg (CD3^+^CD4^+^CD25^high^CD127^−^FOXP3^+^CD39^+^CD73^+^), it detected an increase in the subpopulation of Crohn's patients with nonbiological therapy when compared with biological treatment (Figure 3(a)). This result suggests that nonbiological therapy could suppress the T cell immune response. Furthermore, in the healthy subjects, we could notice an increase in the expression of a subpopulation of Treg that has the CD39 marker absent in the analysis (CD3^+^CD4^+^CD25^high^CD127^−^FOXP3^+^CD39^−^CD73^+^) when compared with Crohn's patients using biologic agents (Figure 3(b)). Deaglio et al. demonstrate that the CD4^+^ T cells maintain the expression of FOXP3 and CD25; without CD39, the CD4^+^ T cells recognize the Treg cell phenotype [18].

Since we observed a higher activation status of CD4^+^ T cells, we decided to stimulate PBMC with PMA/ionomycin. Our results did not show any significant difference in the production of IL-17, TNF-*α*, or IFN-*γ* (data not shown). However, since we detected a higher expression of ROR*γ*t on CD4^+^T cells, we further investigated the ratio of CD4^+^T cells secreting IL-17 and natural Tregs. Crohn's patients on nonbiological therapy show an IL-17/Treg ratio higher than the median value for patients under biological therapy (Figure 3(c)). Natural Treg cells are inversely related to CD4^+^ T cell activation (as measured by HLA-DR^+^ and CD38^+^ expression in CD4^+^T cells) (Figures 3(d) and 3(e)).

### 3.5. Suppressor Treg on Gut Tissue

**Figure 4:**
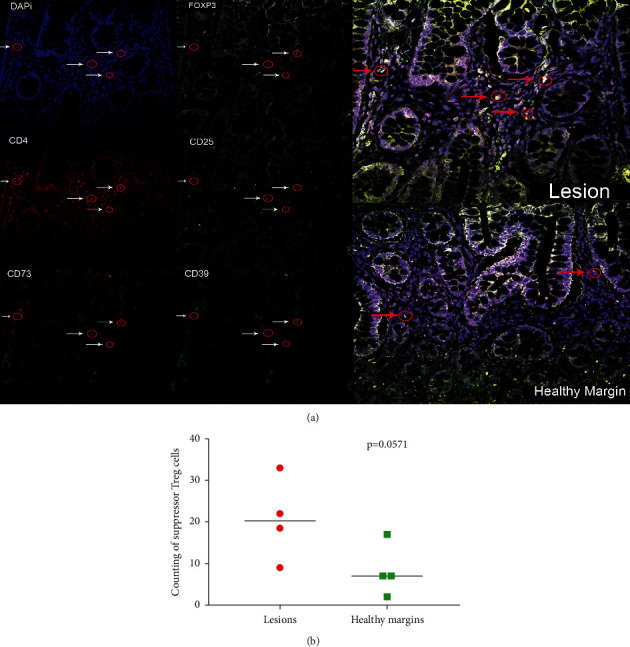
Presence of Treg suppressor on the intestine. (a) Six immunostaining with the specific antibodies. From right to left, we have the merged images. The top right is an example of lesion tissue and bottom-right healthy margins. The six quadrants from the left show an example of a single stain. (b) Counting of suppressor Treg cells in the intestine. Each dot represents the median of the count. Statistical analysis was performed using the Mann–Whitney test.

To address Treg cells' presence on gut tissue, we performed a confocal analysis of four patients before surgery (Figure 4(a)). We observed cells expressing Treg suppressor markers on the lesion area (*p*=0.0571) (Figure 4(b)). The limitation of these results was the reduced number of biopsies that we could have access to. However, we believe that it should be investigated in future studies because it will provide important information about Crohn's pathogenesis and treatment efficacy.

## 4. Discussion

CD4^+^ T cells are responsible for the immune response's critical functions, including the secretion of diverse types of cytokines. CD4^+^ T cells play a pivotal role in CD, as they regulate proinflammatory cytokine production, such as TNF-*α*, an essential mediator for IBD pathogenesis. It has been reported that Crohn's patients have increased levels of CD4^+^ T cells in their gut tissue, especially cells involved in memory immunity, such as CD4^+^CD45RO^+^CD69^+^ [19], even though we observed increased CD4^+^ T cells in both treatment groups. Our data show that the percentage of cells expressing CD4^+^ and CD45RO^+^ expression decreased in TNF-treated patients compared to healthy controls. The same profile was observed when median fluorescence intensity was measured. We believe that the discrepancy between results is due to Bishu and colleagues [19] using tissue samples, whereas we tested blood samples. Additionally, the lower levels of CD4^+^CD45RO^+^ cell percentage compared to increased CD4^+^ T cells in blood may be due to the increased number of circulating naïve cells and more differentiated recruitment of inflamed gut cells.

CD38 is a surface marker most prevalent on cells associated with regulatory activity, such as T regulatory cells and central memory T cells [20]. We have little information about CD4^+^CD38^+^ cells in IBD, but Song and colleagues [21] demonstrated an association of such cells with HIV proliferation and persistence in patients receiving antiretroviral therapy. The authors correlate the expression of CD38 on central memory T cells with increased proliferation and survival, thus rendering cells reservoir of HIV. Furthermore, we observed an increase in CD4^+^CD38^+^ cells in corticoid-treated patients compared to healthy controls, while TNF-treated patients showed decreased levels. These two distinct profiles suggest that despite corticoids being capable of inducing remission, they do not increase T cells' capacity to respond to newer antigenic stimulation [17, 22, 23]. At the same time, anti-TNF-*α* can reduce central memory T cells. Furthermore, vitamin D blood levels are known to be correlated with increased expression of CD38-expressing CD4 T cells [24]. Nevertheless, it is noteworthy that IBD patients suffer from vitamin D deficiency [25]; thus, the two distinct profiles observed may be influenced by such vitamin deficiency.

Since we had alterations in the CD4 T cells and the activation markers, the question of who is regulating the immune response in patients arises? Moreover, if Treg cells can control the dysregulated immune response? Our results demonstrate no difference in the Treg (CD4^+^CD25^+^FOXP3^+^) blood levels, showing similar results as Grundstrom and colleagues [26]. However, previous reports [27, 28] showed the opposite results; IBD TNF-treated patients who responded to monoclonal antibody treatment had increased blood levels of Treg. This difference could be related to the methodology, and the cohort has colitis and Crohn's patients. The induction of CD39 expression constitutes a common immunoregulatory mechanism triggered by STAT3-activating cytokines in the innate immune system [29, 30]. In addition, CD39 mediates the suppressive activity of mouse and human Treg cells, probably through the generation of adenosine [18, 31–33]. CD39 is responsible for scavenging proinflammatory molecules and producing immunosuppressive ones. Mice expressing higher proportions of human CD39^+^ and CD73^+^cells showed attenuated symptoms from DSS-induced colitis [34]. Our results showed fewer CD4^+^CD25^+^CD73^+^CD39^−^FOXP3^+^ cells in TNF-treated patients than in healthy controls. These data indicate a shift in cell proportions due to the recruitment of anti-inflammatory cells to the inflamed gut. The confocal analysis confirmed the presence of Treg suppressor cells in the lesion area, suggesting that anti-TNF might induce an anti-inflammatory profile.

Furthermore, a higher Th17/Treg cell ratio was associated with nonbiological treatment subjects. In addition, we detected a negative correlation between Treg cells and CD4^+^ T cells activation markers (HLA-DR and CD38). These data suggest that the biological treatment could improve Treg function and the Th17/Treg cell ratio associated with reducing chronic inflammation.

## 5. Conclusion

The treatment of Crohn's disease in developing countries like Brazil is a clinical challenge for clinicians and researchers. Understanding how biological therapies work versus conventional therapy is essential to improving the quality of life, prognosis, and decreasing disease relapse. Here, we were able to show the primary treatment used in Brazil for Crohn's patients in the context of CD4^+^ T cell immune response. Although our data do not bear directly on the efficiency of treatments, they indicate that patients undergoing anti-TNF therapy show an immune response similar to healthy individuals.

## Figures and Tables

**Table 1:** Demographic, clinical, and laboratory characteristics.

	Healthy subjects	Crohn's patients		*P* value
		Biological	Nonbiological	
Case number	28	25	16	
Treatment				
	None	Adalimumab (*n* = 21) Infliximab (*n* = 4)	Anti-inflammatory + immunosuppressive + corticoids (*n* = 5) Anti-inflammatory + immunosuppressive (*n* = 4) Anti-inflammatory + corticoids (*n* = 6) Anti-inflammatory (*n* = 1)	
Gender (%)				
Female	68%	72%	69%	
Male	32%	28%	31%	

Age (years)	39	36	41	
Calprotectin (*μ*g/g)	55.4: 36–107	245: 144–631	422: 89.6–1014	Healthy vs. nonbiological, *p*=0.0003 Healthy vs. biological, *p*=0.0003
MCV (mean corpuscular volume) (fl) Median: IQR	83.9: 82.13–87.1	86.8: 83.4–90.6	87.15: 85.2–91.18	Healthy vs. nonbiological, *p*=0.0394
				
CHCM (cellular hemoglobin concentration mean) (g/dL) Median: IQR	34.65: 34.3–34.75	34.3: 33.15–34.75	33.13: 32.5–33.65	Healthy vs. nonbiological, *p*=0.0018
RDW (red cell distribution width) (%) Median: IQR	12.6: 12.33–13.05	13.2: 12.75–13.85	13.2: 12.9–13.38	Healthy vs. biological, *p*=0.0214
Albumin (g/dL) Median: IQR	4.4: 4.2–4.575	4.2: 4–4.4	4: 3.85–4.2	Healthy vs. nonbiological, *p*=0.0007
Globulin (g/dL) Median: IQR^*∗*^	2.9: 2.8–3.15	3.1: 2.9–3.55	3.1: 3–3.175	Healthy vs. biological, *p*=0.0192
ASCA_IgG (U/m) Median: IQR	5.6: 2.3–9.6	15.9: 9.8–35.5	11.6: 3.4–28.7	Healthy vs. biological, *p*=0.0006
ASCA_IgA (U/m) Median: IQR	0.9: 0.5–2.2	6.4: 2.0–15.8	2.25: 1.1–3.3	Healthy vs. biological, *p* < 0.0001

^*∗*^IQR, interquartile, Kruskal–Wallis statistics.

## Data Availability

Due to ethical concerns, clinical and laboratory data cannot be made openly available and are available from the corresponding author upon request.

## References

[1] Kaplan G. G., Windsor J. W. (2021). The four epidemiological stages in the global evolution of inflammatory bowel disease. *Nature Reviews Gastroenterology & Hepatology*.

[2] Ng S. C., Shi H. Y., Hamidi N. (2017). Worldwide incidence and prevalence of inflammatory bowel disease in the 21st century: a systematic review of population-based studies. *The Lancet*.

[3] Cosnes J., Gower-Rousseau C., Seksik P., Cortot A. (2011). Epidemiology and natural history of inflammatory bowel diseases. *Gastroenterology*.

[4] Hammer T., Langholz E. (2020). The epidemiology of inflammatory bowel disease: balance between East and West? A narrative review. *Digestive Medicine Research*.

[5] Parente J. M. L., Coy C. S., Campelo V. (2015). Inflammatory bowel disease in an underdeveloped region of Northeastern Brazil. *World Journal of Gastroenterology*.

[6] Quaresma A. B., Kaplan G. G., Kotze P. G. (2019). The globalization of inflammatory bowel disease. *Current Opinion in Gastroenterology*.

[7] Argollo M., Kotze P. G., Kakkadasam P., D’Haens G. (2020). Optimizing biologic therapy in IBD: how essential is therapeutic drug monitoring?. *Nature Reviews Gastroenterology & Hepatology*.

[8] Ananthakrishnan A. N. (2015). Epidemiology and risk factors for IBD. *Nature Reviews Gastroenterology & Hepatology*.

[9] Cui G., Yuan A. (2018). A systematic review of epidemiology and risk factors associated with Chinese inflammatory bowel disease. *Frontiers of Medicine*.

[10] Fontenot J. D., Gavin M. A., Rudensky A. Y. (2003). Foxp3 programs the development and function of CD4+CD25+ regulatory T cells. *Nature Immunology*.

[11] Sasson S. C., Slevin S. M., Cheung VT. (2021). Interferon-gamma-producing CD8+ tissue resident memory T cells are a targetable hallmark of immune checkpoint inhibitor-colitis. *Gastroenterology*.

[12] Schorer M., Lambert K., Rakebrandt N. (2020). Rapid expansion of treg cells protects from collateral colitis following a viral trigger. *Nature Communications*.

[13] Bynoe M. S., Viret C. (2008). Foxp3^+^ CD4^+^ T cell-mediated immunosuppression involves extracellular nucleotide catabolism. *Trends in Immunology*.

[14] Tippalagama R., Singhania A., Dubelko P. (2021). HLA-DR marks recently divided antigen-specific effector CD4 T cells in active tuberculosis patients. *The Journal of Immunology*.

[15] Tincati C., Bellistrì G. M., Ancona G., Merlini E., d’Arminio Monforte A., Marchetti G. (2012). Role of *in vitro* stimulation with lipopolysaccharide on T-cell activation in HIV-infected antiretroviral-treated patients. *Clinical and Developmental Immunology*.

[16] Mickael M. E., Bhaumik S., Basu R. (2020). Retinoid-related orphan receptor ROR*γ*t in CD4^+^ T-cell-mediated intestinal homeostasis and inflammation. *American Journal of Pathology*.

[17] Sallusto F., Geginat J., Lanzavecchia A. (2004). Central memory and effector memory T cell subsets: function, generation, and maintenance. *Annual Review of Immunology*.

[18] Deaglio S., Dwyer K. M., Gao W. (2007). Adenosine generation catalyzed by CD39 and CD73 expressed on regulatory T cells mediates immune suppression. *Journal of Experimental Medicine*.

[19] Bishu S., el Zaatari M., Hayashi A. (2019). CD4^+^ tissue-resident memory T cells expand and are a major source of mucosal tumour necrosis factor alpha in active Crohn’s disease. *Journal of Crohn’s and Colitis*.

[20] van de Donk N. W. (2018). Immunomodulatory effects of CD38-targeting antibodies. *Immunology Letters*.

[21] Song C. B., Zhang L. L., Wu X. (2020). CD4(+)CD38(+) central memory T cells contribute to HIV persistence in HIV-infected individuals on long-term ART. *Journal of Translational Medicine*.

[22] Gehad A., Teague J. E., Matos T. R. (2018). A primary role for human central memory cells in tissue immunosurveillance. *Blood Advances*.

[23] Jameson S. C., Masopust D. (2018). Understanding subset diversity in T cell memory. *Immunity*.

[24] Villegas-Ospina S., Aguilar-Jimenez W., Gonzalez S. M., Rugeles M. T. (2017). Vitamin D modulates the expression of HLA-DR and CD38 after in vitro activation of T-cells. *Hormone Molecular Biology and Clinical Investigation*.

[25] Gubatan J., Moss A. C. (2018). Vitamin D in inflammatory bowel disease: more than just a supplement. *Current Opinion in Gastroenterology*.

[26] Grundstrom J., Linton L., Thunberg S. (2012). Altered immunoregulatory profile during anti-tumour necrosis factor treatment of patients with inflammatory bowel disease. *Clinical and Experimental Immunology*.

[27] Guidi L., Felice C., Procoli A. (2013). FOXP3^+^T regulatory cell modifications in inflammatory bowel disease patients treated with anti-TNF*α*Agents. *BioMed Research International*.

[28] Clough J. N., Omer O. S., Tasker S., Lord G. M., Irving P. M. (2020). Regulatory T-cell therapy in Crohn’s disease: challenges and advances. *Gut*.

[29] Mascanfroni I. D., Yeste A., Vieira S. M. (2013). IL-27 acts on DCs to suppress the T cell response and autoimmunity by inducing expression of the immunoregulatory molecule CD39. *Nature Immunology*.

[30] Zhou F., Zhang G.-X., Rostami A. (2018). Distinct role of IL-27 in immature and LPS-induced mature dendritic cell-mediated development of CD4+ CD127+3G11+ regulatory T cell subset. *Frontiers in Immunology*.

[31] Schneider E., Winzer R., Rissiek A. (2021). CD73-mediated adenosine production by CD8 T cell-derived extracellular vesicles constitutes an intrinsic mechanism of immune suppression. *Nature Communications*.

[32] Gandhi R., Kumar D., Burns E. J. (2010). Activation of the aryl hydrocarbon receptor induces human type 1 regulatory T cell-like and Foxp3(+) regulatory T cells. *Nature Immunology*.

[33] Gutiérrez-Vázquez C., Quintana F. J. (2018). Regulation of the immune response by the aryl hydrocarbon receptor. *Immunity*.

[34] Robles R. J., Mukherjee S., Vuerich M. (2020). Modulation of CD39 and exogenous APT102 correct immune dysfunction in experimental colitis and Crohn’s disease. *Journal of Crohn’s and Colitis*.

